# Influence of a 23S ribosomal RNA mutation in *Helicobacter pylori* strains on the *in vitro* synergistic effect of clarithromycin and amoxicillin

**DOI:** 10.1186/1756-0500-5-603

**Published:** 2012-10-30

**Authors:** Türkan Sakinc, Barbara Baars, Nicole Wüppenhorst, Manfred Kist, Johannes Huebner, Wolfgang Opferkuch

**Affiliations:** 1 Division of Infectious Diseases, University Hospital Freiburg, Hugstetter Strasse 55, Freiburg, D-79106, Germany; 2 Department of Medical Microbiology, Ruhr-University Bochum, Universitätstraße 150, Bochum, 44801, Germany; 3 Institute of Medical Microbiology and Hygiene, National Reference Centre for Helicobacter, University Medical Centre Freiburg, Freiburg, Germany

**Keywords:** *Helicobacter pylori*, Synergistic effect, 23S rRNA

## Abstract

**Background:**

Clarithromycin (CLR) is the most commonly recommended antibiotic in *Helicobacter pylori* eradication regimens, but the prevalence of CLR-resistant *H*. *pylori* is increasing. CLR resistance is associated with mutations in the 23S rRNA gene. However, *H. pylori* eradication can still be achieved with triple therapy, and an additive effect may occur with multiple antibiotics.

**Methods:**

Twenty-six CLR-resistant strains were examined. The MIC of clarithromycin was determined by agar-dilution-testing on Columbia agar, as described elsewhere. The conserved region of the H. pylori 23S rRNA gene between nucleotide positions 1445 and 2846 [GenBank: U27270] was amplified. RFLP and sequence analysis were performed with the 1402-bp PCR product. Synergy between clarithromycin and amoxicillin was assessed using the agar dilution checkerboard technique. To confirm the correlation between mutation and synergistic effect with subinhibitory concentrations of AMX, site-directed mutagenesis was performed in four CLR-susceptible H. pylori isolates.

**Results:**

Twenty-six clarithromycin-resistant strains were examined. The conserved region of the *H. pylori* 23S rRNA gene was amplified, and the purified PCR product was checked for mutations by restriction fragment length polymorphism (RFLP) analysis and sequencing. A synergistic effect was found in only three of the 12 *H. pylori* strains (25%) with the A2142G mutation and five of the 10 *H. pylori* strains (50%) with the A2143G mutation (fractional inhibitory concentration: FIC < 0.5, minimal inhibitory concentration: MIC<2 mg/L) was found. Site-directed mutagenesis was performed in four CLR-susceptible *H. pylori* isolates.

Three of these isolates harboring a mutation in position A2143G grew under selection with CLR (MIC >16 mg/L), and all three strains showed the synergistic effect (FIC<0.5). In contrast, three of the same four strains transformed with DNA fragments with a mutation in position A2142G were resistant to CLR (MIC>16 mg/L) and showed no synergism with amoxicillin (FIC>2).

**Conclusions:**

Here we demonstrate that in 100% of the *in vitro* transformed strains, a mutation at position A2143G leads to a synergistic effect between clarithromycin and amoxicillin, whereas a mutation at position at A2142G had no discernible effect.

## Background

Infection with *H. pylori* is the major cause of chronic gastritis and peptic ulceration and an important factor in the development of gastric cancer [1]. The introduction of triple therapy (i.e., the combination of two antibiotics and a proton pump inhibitor) was a major advance in the treatment of gastritis and peptic ulcer disease [2,3]. However, the initial success of such therapy was blunted by the emergence of resistance against the first-line drugs clarithromycin (CLR) and metronidazole (MTZ) [2,3].

The major cause of CLR resistance in *H. pylori* strains is point mutations in domain V of the 23S ribosomal RNA gene (A2142G, A2143G) [3,4]. An earlier publication identifies two different CLR-resistant populations: one with an A2142G mutation and MIC values >16mg/L; and the other with an A2143G mutation and MIC values of 8-16mg/L [5].

Surprisingly, *H. pylori* eradication can still be achieved with triple therapy (CLR, amoxicillin/AMX), and a proton pump inhibitor) even if the strain tests CLR- resistant [6]. This points to a synergistic effect between CLR and AMX. To study this synergism, we tested 26 CLR-resistant *H. pylori*-strains [7]*in vitro* with the checkerboard method and subsequently correlated the results with mutations in the 23S ribosomal RNA gene.

## Methods

### Bacterial strains and media

Twenty-six clinical CLR-resistant isolates of *H. pylori* were tested [7]. The strains were originally obtained from gastric biopsy samples and were kindly provided by B. Baars [7]. In addition, the following four CLR-susceptible *H. pylori* strains were used for mutagenesis and resistance experiments: strain CCUG 38772 (which contains a mutation at position A2142G in the 23S rRNA gene) and strain 30K 184 (with a mutation at position A2143G) were obtained from the German national reference centre for *H. pylori* (Freiburg, Germany) and used as control strains. Brain heart infusion bouillon (BHI, Oxoid CM225), supplemented with 1% hemin and 10% horse serum, was used for liquid culture, whereas columbia agar (Oxoid CM 331) supplemented with 10% sheep- blood (Oxoid FSR 1055) was used as solid medium.

### Susceptibility testing

The MIC of clarithromycin was determined by agar-dilution-testing on Columbia agar, as described elsewhere. The plates were incubated at 37°C under microaerophilic conditions (i.e., 5% CO_2_) for 5–7 d. Bacterial strains were classified as clarithromycin resistant if the MIC was ≥1 mg/L.

### Synergy testing

Synergy between clarithromycin and amoxicillin was assessed using the agar dilution checkerboard technique. For quantitation of synergism, the fractional inhibitory concentration (FIC) indices for both combinations were calculated as described by Eliopoulos et al. [8]. The ranges used for description of the FIC indices were as follows: <0.5, synergy; > 0.5 to 1, additive; >1 to 4, indifference; >4, antagonism.

### DNA isolation and PCR of 23S rRNA gene

Genomic DNA was isolated using the QIAamp DNA mini kit (Qiagen, Hilden, Germany) according to the manufacturer′s instructions. The conserved region of the *H. pylori* 23S rRNA gene between nucleotide positions 1445 and 2846 [GenBank: U27270] was amplified using forward primer 5′- AGTCGGGGACCTAAGGCGAG - 3′- and reverse primer 5′ - TTCCCGCTTAGATGCTTTCAG- 3′ with the genomic DNA of 26 CLR-resistant *H. pylori* isolates serving as a template [5].

### RLFP analysis

RFLP analysis was performed with the 1402-bp PCR product obtained as described above. To detect the A-to-G point mutation at nucleotide position 2142, the DNA was digested with restriction enzymes *Bbs*I, and for detection of the mutation at position 2143, digestion with *Bsa*I was used. The resulting DNA fragments were then analyzed by electrophoresis on 0.8% agarose gels.

### DNA sequencing and sequence analysis

PCR fragments were purified using the QIAqiuck PCR Purification Kit (Qiagen, Hilden, Germany). Sequencing was performed using the BigDye Terminator v3.1 sequencing kit and analyzed on an ABI PRISM 377 Genetic Analyzer. The sequences were analyzed using free ClustalW software.

### Site-directed mutagenesis

To construct an A2142G mutation, fragments were amplified with primer pairs DP1 (5′ACGGCGGCCGTAACTATA 3′) /GW11 (5′GGTCTTCCCGTCTTGCCGC 3′) and ZGE23 (5′ACAGGCCAGTTAGCTA 3′) /GW1 (5′GACGGGAAGACCCCGTGGA 3′), and for A2143G, mutation fragments were amplified with primer pairs DP1/GW14 (5′GGTCTCTCCGTCTTGCCGC 3′) and ZGE23 /GW4 (5′GACGGAGAGACCCCGTGGA 3′) as described by Ge and Taylor [5]. The final 307-bp fragments of all four strains were confirmed by nucleotide sequencing and then introduced into the CLR-susceptible *H. pylori* isolates by natural transformation as described previously [5]. Grown colonies (transformants) on CLR agar plates after four days were confirmed for successful transformation and mutation by PCR, followed by DNA sequencing analysis. In addition, from these positive colonies whole RNA for a reverse transcription PCR assay was isolated to control RNA expression and mutation in the 23S rRNA gene.

## Results and discussion

Twenty-six CLR-resistant strains were examined by RLFP (data not shown) and sequencing. This analysis revealed that 12 of the *H. pylori* isolates carried the A2142G mutation, and an additional 10 *H. pylori* isolates harbored the A2143G mutation (Table 1). Four CLR-resistant *H. pylori* isolates did not display any mutation. Only three of the 12 *H. pylori* strains (25%) with the A2142G mutation and five of ten *H. pylori* strains (50%) with the A2143G mutation showed a synergistic effect (FIC < 0.5, MIC<2 mg/L) with subinhibitory concentrations of AMX. These results suggest that the synergistic effect might also depend on the mutation at position A2143G in the 23S rRNA gene.

**Table 1:** **Summary of checkerboard results: 26 *H. pylori *strains tested with clarithromycin versus amoxicillin correlated to mutations in 23S rRNA**

**Mutation in 23S rRNA gene**	**No. of strains**	**Synergy (FIC < 0.5)**	**MIC**
A2143G	10	5 (50%)	<2
A2142G	12	3 (25%)	>16

FIC: fractional inhibitory concentration [8].

To confirm the correlation between mutation and synergistic effect with subinhibitory concentrations of AMX, site-directed mutagenesis was performed in four CLR-susceptible *H. pylori* isolates. Each point mutation at position A2142G or A2143G of the *H. pylori* 23S rRNA gene was generated by a sequential PCR method [5] using genomic DNA from four CLR-susceptible *H. pylori* strains as a template for each amplification. The obtained transformants were controlled/verified for point mutation as described under 'Methods'.

As shown in Table 2, three of four strains transformed with DNA fragments harboring a mutation in position A2143G grew under selection with CLR (MIC >16 mg/L). All three strains showed a synergistic effect (FIC<0.5) when combined with subinhibitory concentrations of AMX. In contrast, though three of four strains transformed with DNA fragments including a mutation in position A2142G did grow with CLR (MIC>16 mg/L), they showed no synergistic effects (FIC>2). One of the four strains was not transformable with either of the two point mutations. These results confirm our assumption that a point mutation in position A2143G may lead to a synergistic effect for AMX and CLR in *H. pylori in vitro* experiments. In other words, a point mutation at position A2142G interferes with or suppresses the synergism.

**Table 2:** ***In vitro *site-directed mutagenesis of *H. pylori *23S rRNA gene and geno- and phenotypes of the mutants obtained**

**No. of strains CLR-susceptible**	**Donor DNA for mutagenesis (region of the 23S rRNA gene)**	**No. of transformants obtained with mutation in 23S rRNA gene**	**Final MIC (CLR)**	**No. of strains showing synergy**	**FIC**
4	A2142G	3 (A2142G)	>16	0 (0%)	>2
	A2143G	3 (A2143G)	>16	3 (100%)	<0.5

Additional experiments have been carried out with the same four CLR-susceptible *H. pylori*-strains used for mutagenesis by incubating them with subinhibitory concentrations of CLR. The MIC increased in these strains to >16mg/L after not more than four passages, but no mutation at the described positions of the 23S rRNA gene could be found, and interestingly, they also demonstrated no synergism. This result clearly confirmed our hypothesis. Accordingly, our data strongly suggest that a mutation at position A2143G may leads to a synergism between AMX and CLR *in vitro*. Furthermore, these results also demonstrate that different mechanisms are responsible for the CLR resistance.

The mutation at A2143G in the 23S rRNA is frequently found in several countries: e.g., Spain 79.4%, Brazil 74%, and Tunisia 88% [9]. It is the dominant mutation in macrolide-resistant strains of *H. pylori*. Besides the resistance loci A2142G and A2143G, several other less frequent resistance loci have been described, such as 23S rRNA mutations at positions 2058 and 2059, which are found in various geographic locations (including Japan) but have not yet been detected in European *H. pylori* isolates [10]. Mutation at T2182C was found in 5.9% of isolates in Spain [9]. It is also known that in addition to mutations in the 23S rRNA gene, other mechanisms are important in CLR resistance *in vivo*, in particular an active multidrug efflux mechanism of *H. pylori*[11].

## Conclusions

Twenty-six CLR-resistant strains were examined. The conserved region of the *H. pylori* 23S rRNA gene was checked for mutations. In addition, site-directed mutagenesis was performed in four CLR-susceptible *H. pylori* isolates. The data show that a mutation at position A2143G in 100% of the *in vitro* transformed strains leads to a synergistic effect between clarithromycin and amoxicillin, whereas a mutation at position at A2142G has no effect in vitro.

## Competing interests

None of the authors of this paper has a financial or personal relationship with other people or organizations that could inappropriately influence or bias the content of the paper.

## Authors’ contributions

ST and BB performed the experimental work. ST, BB, and WO coordinated the study and edited the manuscript. NW, MK, and JH helped to edit the manuscript. All authors read and approved the final manuscript.
